# Effect of nonuniform perpendicular anisotropy in ferromagnetic resonance spectra in magnetic nanorings

**DOI:** 10.1038/s41598-021-93597-8

**Published:** 2021-07-09

**Authors:** E. Saavedra, A. Riveros, J. L. Palma

**Affiliations:** 1 Departamento de Física, Universidad de Santiago de Chile, 9170124 Santiago, Chile; 2 Escuela de Ingeniería, Universidad Central de Chile, 8330601 Santiago, Chile; 3 Center for the Development of Nanoscience and Nanotechnology (CEDENNA), 9170124 Santiago, Chile

**Keywords:** Physics, Condensed-matter physics, Magnetic properties and materials

## Abstract

The high frequency dynamic behaviors of magnetic nanorings with variable anisotropy along their radius have been studied using micromagnetic simulations. The dynamic susceptibility spectrum and spatial localization of the ferromagnetic resonance modes are investigated by varying anisotropy gradients in nanorings of 200 nm of external radius, with different internal radii. Both the resonant frequencies and the number of peaks depend on the lower energy magnetization configuration which in turn is a function of anisotropy gradients. Besides, it is shown that the effects of the anisotropy gradient are relevant even for the narrowest ring of 10 nm wide. The idea of controlling frequencies by modifying the anisotropy gradients of the system suggests the possibility of using these nanostructures in potential magnetic controllable frequency devices.

## Introduction

Magnetic nanostructures have generated great interest in the last years, mainly due to the experimental techniques’ progress, which allow to obtain nanometer length scales nanostructures in two and three dimensions. The basic scientific interest in the magnetic properties of these systems, allows us to know and control the magnetic behavior which can be used for the production of magnetic devices, such as high-density storage media^1, 2^, high-speed magnetic random access memories^3, 4^, magnetic sensors^5^, high frequency oscillators^6, 7^ and logic devices^8, 9^.

Moreover, there is a new spectrum of applications in the lithographed magnetic nanostructures by exploring their magnetic dynamical properties^10^, allowing the control of their properties by tailoring their geometry^11^. Among the available geometries, magnetic nanorings have attracted a lot of attention as promising structures for devising new and effective microwave absorbers and since they can be used as efficient magnetic nanoagents in systematic cancer treatment by magnetothermodynamic therapy^12^. Nanorings are described by its height and external and internal radii, so they offer one more geometrical parameter to get control compared with the nanodots. More important, they offer a stable flux-closure magnetization pattern, where the magnetization follows the ring edges with a core-free magnetic configuration which allows a most reproducible switching field to be used in applications^13^.

Planar nanorings have a magnetization easy axis in the plane of the nanostructure, allowing a vortex-kind reversal. If we include a nonuniform out of plane magnetic anisotropy this allows new magnetic configurations with different topological magnetic textures, like knot and meron^14^. These new kinds of magnetic textures that appear, depend on the radial uniaxial perpendicular anisotropy strength and allow different dynamic of the magnetization and band-gaps in ferromagnetic resonance (FMR) spectra. It has been shown that such nonuniform magnetic anisotropy can be controlled, for instance, by temperature^15^ and electric field^16^ or ion implantation^17^. For recording applications, quasi–static studies are needed in order to know the stability for every configuration depending on the geometry^14^. Moreover, for sensors and oscillators applications, dynamic characterization of these configurations are required. For these reasons, in this work we explore the dynamic response of the magnetic stables configurations for a nanoring, such as: knot, meron and vortex, by analyzing the FMR spectra. The studied geometries for magnetic nanorings are external radius of 200 nm and with a thickness of 10 nm, with different internal radii and including a non uniform radial perpendicular anisotropy gradient. These geometrical parameters were chosen in accordance with the work of Castro et al.^14^. Micromagnetic simulations where used in order to study the FMR spectra of these kind of magnetic nanostructures, by solving the Landau Lifshitz Gilbert (LLG) equation and using the Ringdown method^18–20^ to obtain the dynamic response of the system. It is worthwhile to mention that dynamical susceptibility using micromagnetic simulations in ring shaped nanostructures was previously extensively studied^21–24^, showing the interest of the scientific community in this topic. Up today, there are no studies about the dynamic response of these geometries when a nonuniform perpendicular anisotropy is included, which is very interesting due to the knot configuration offer a non symmetric configuration of the magnetization which offer more control parameters for the dynamic response, and the meron configuration contains an out-of-plane component of the magnetization thats allows to appear new resonant modes. In that way in this paper we study in detail the modes and resonant peaks as a function of the non uniform perpendicular anisotropy strength and ring aspect ratio.

## Micromagnetic simulations

We have performed micromagnetic simulations using the OOMMF code^25^ to solve the magnetization dynamic $\mathbf {M}(t)$ given by the LLG equation^26, 27^: $\displaystyle {\frac{d\mathbf {M}}{dt} = -\gamma \mathbf {M} \times \mathbf {H}_\text {eff} + \frac{\alpha }{M_s} \mathbf {M} \times \frac{d\mathbf {M}}{dt} }$, where $\gamma$, $M_s$ are the gyromagnetic ratio and the saturated magnetization value, respectivly, while $\alpha$ is the damping coefficient. We have set $\alpha = 0.015$ which is a ten times below the typical values used for dynamical simulations^28^ and allows to get a better resolution of the spin wave modes in the spectra. We have considered a magnetic nanoring of 10 nm of thickness and external radius $R_2 = 200$ nm, with different internal radii $R_1 = 50, 100, 160$ and 190 nm. The magnetic magnetic parameters were extracted from Ref.^29^, i.e. saturation magnetization $M_s= 860$ kA/m, exchange length $l_\text {ex} =5.08$ nm. Besides, the nonuniform perpendicular magnetic anisotropy was included by a radial profile extracted from Ref.^14^:

1 $$\begin{aligned} K(r) = K \frac{(r-R_1)}{(R_1-R_2)} , \quad R_1 \le r \le R_2 \end{aligned}$$

where *r* is the radial distance from the ring center and $K \ge 0$ is the Radial Perpendicular Magnetic Anisotropy (RPMA) strength. Note that when $r = R_2$ (external border of the ring) *K*(*r*) is maximum, the minus sign ensures that the magnetization is energetically favored along the perpendicular axis of the ring plane, while when $r = R_1$ (internal border of the ring) $K(r) = 0$.

We have studied the magnetic configurations of the nanoring, for the four internal radii sizes, as a function of the RPMA strength *K* in the range $0\le K \le 500\,\hbox {kJ}/\hbox {m}^3$ by steps of $10\, \hbox {kJ}/\hbox {m}^3$. It is worthwhile to mention that perpendicular anisotropy strengths of $1 \,\hbox {MJ}/\hbox {m}^3$ can be obtained experimentally by epitaxial growth in L1$_0$ materials^30^ even for thin films of 10 nm of thickness^31^. Moreover, for each stable magnetic configuration we have analyzed the magnetic response by FMR obtaining the resonance modes of the frequency spectrum using a Ringdown method^18^, generated by an exponential pulse which rapidly decays in time, $h(t) = 1000 \exp {(-10^9t)}\,\hbox {A/m}$^21, 32–36^. The pulse was directed along the *x*-direction (in the nanoring plane). Nevertheless, for the knot state we have also analyzed the effect of the magnetic pulse for different in plane directions (due to the loss of the azimuthal symmetry of this magnetic state). Note that the excitation amplitude of 1000 A/m is small enough to remain in the linear response region^33, 37^. The magnetization was stored for 4 ns (4000 time steps) at uniform time intervals of 1 ps. The short pulse excites the magnetization along the pulse direction and the frequency spectrum is obtained by calculating the dynamical susceptibility using Fast Fourier Transform (FFT). The sample frequency (resolution) was 0.25 GHz.

## Results and analysis

In this section, we show and analyze the results of the micromagnetic simulations for both the static and dynamic properties of the nanoring for different aspect ratio and RPMA strength *K*-values. For the static behavior we focus on the stable magnetic configurations at zero field for different nanoring internal radii. On the other hand, for the dynamics we focus on the dynamic susceptibility and the resonance frequency peaks of a short in-plane magnetic pulse as a function of the ring aspect ratio and RPMA strength. We also study the spatial distribution of the dynamic susceptibility. Besides, it is expected that the RPMA profile has influence in wider rings, but is not clear if the RPMA has a significant difference compared with the uniform distributed Perpendicular Magnetic Anisotropy (PMA) for the narrowest ring. For this reason, we also compare the effect of RPMA profile on the resonance spectrum respect to a uniform PMA, in order to clarify the effects of RPMA on narrower nanorings.

### Static magnetic properties

**Figure 1:**
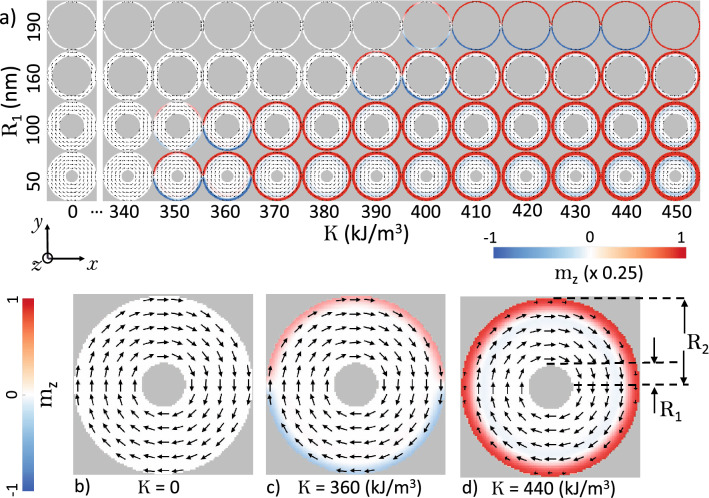
(**a**) Magnetic ground states for the magnetic nanoring for different values of the RPMA strength K and for different values of the ring internal radius (for a visual purpose the color scale for $m_z$ was rescaled by a factor 0.25). Typical  magnetic states for a wide ring ($R_1= 50 \,\hbox {nm}$), (**b**) a vortex state, (**c**) a knot state and (**d**) a meron state. In (**b**–**d**) the color scale for $m_z$ was not rescaled.

We start the analysis by studying the stable magnetic configurations for different nanorings, from a wide nanoring with internal radius of 50 nm, up to a narrow nanoring with internal radius of 190 nm. The convergence states of the micromagnetic simulations at zero field for different values of the RPMA strength *K* and length of the internal radius $R_1$ of the nanoring are shown in Fig. 1a. The magnetization component along the perpendicular direction to the ring plane $m_z = M_z/M_s$ is shown by a color scale, while the in plane magnetization direction are depicted by black arrows. For a visual purpose in Fig. 1a we have re-scaled $m_z$ by a factor 0.25. As it can be seen, depending of the strength of the RPMA, there exist 3 non trivial magnetic states: a vortex state (a curling in plane magnetic state), a knot state (a curling magnetic state with perpendicular magnetization in the ring external edge without azhimutal symmetry) and a meron state (a curling magnetic state with perpendicular magnetization in the ring external edge with azhimutal symmetry), which agree with Ref.^14^.

For example for $R_1 = 50$ nm (the wider ring), the vortex state is stable until $K = 340\,\hbox {kJ}/\hbox {m}^3$, while for higher *K*-values due to the RPMA the curling in plane magnetization still holds except close the external edge of the nanoring (where the RPMA strength is maximum), leading to nonzero magnetization component perpendicular to the ring plane at the ring external edge, stabilizing a knot (for $K = 350$ and $360 \,\hbox {kJ}/\hbox {m}^3$) and meron magnetic states (for higher values of *K*). Note as the ring gets narrower the vortex is stable until higher *K*-values, for example for the narrowest ring ($R_1 = 190$ nm) the vortex is stable until $K = 390 \,\hbox {kJ}/\hbox {m}^3$, then for higher *K*-values the knot state lives until $K = 440 \,\hbox {kJ}/\hbox {m}^3$, and the meron state not appear, even for higher values than $K = 450\, \hbox {kJ}/\hbox {m}^3$, since to in the narrow magnetic region of the ring there are not enough magnetic material to host a meron, the magnetic state converge to a uniformly magnetized ring along the z-direction. For a sake of clearness, in Fig. 1 b,c,d we show these 3 magnetic states for the case $R_1 = 50\,\hbox {nm}$ without rescaling the $m_z$-magnetization component.

### Dynamic magnetic properties

**Figure 2:**
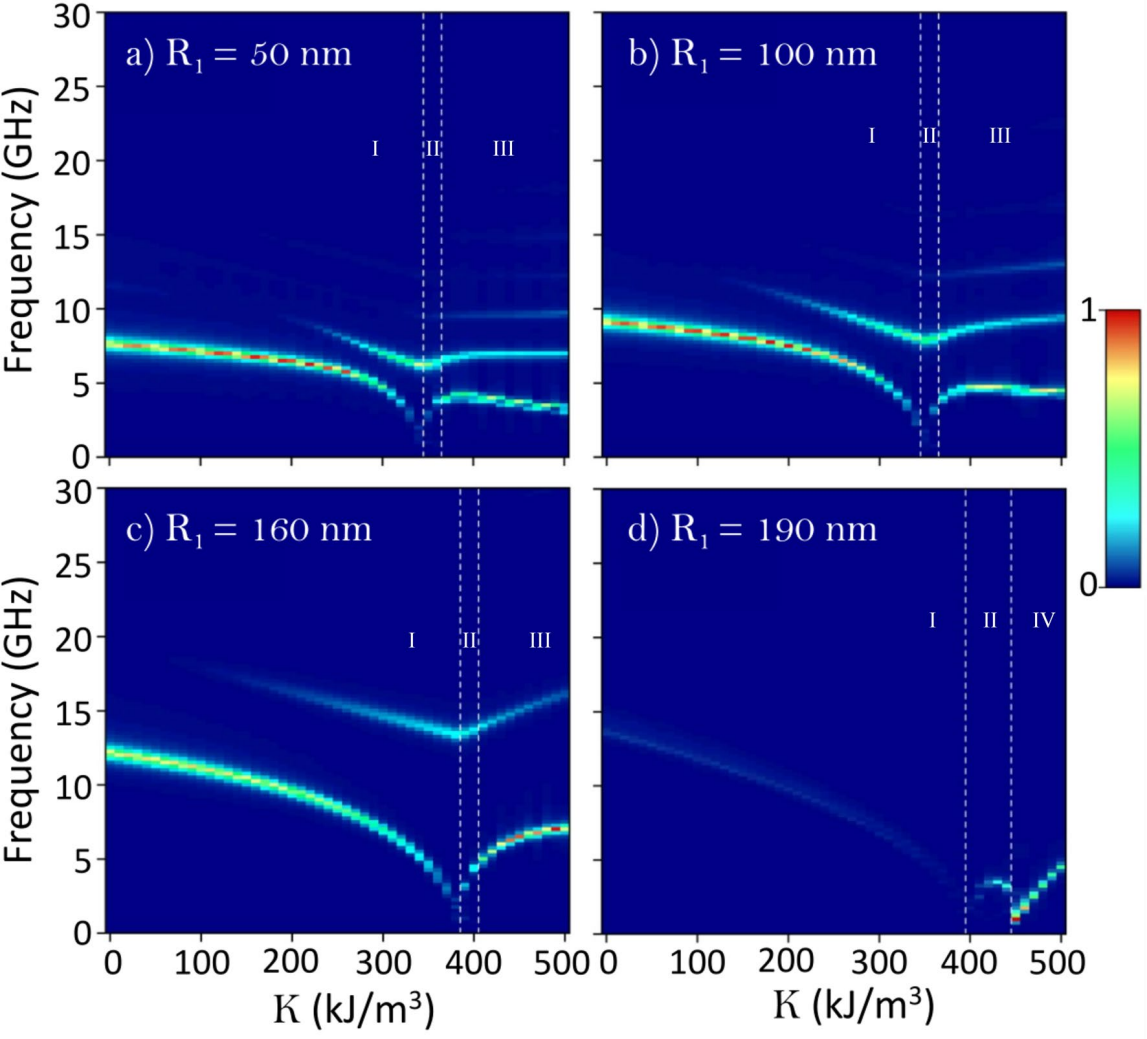
(**a**) Imaginary component of the dynamic susceptibility for a pulse in the *x*-direction as a function of the sample frequency and the RPMA strength *K*, for the magnetic nanoring with internal radii (**a**) $R_1 = 50$, (**b**) 100, (**c**) 160 and (**d**) 190 nm. In each figure the lines (curves) with highest amplitude for the dynamical susceptibility correspond to the main modes of resonance. The depicted regions I,II, III and IV correspond to the regions where the vortex, knot, meron and uniform out-of-plane magnetic states, respectively, are stable in the nanoring.

We continue this study by analyzing the dynamic magnetic response of the nanoring due to the exciting in-plane magnetic pulse (the details of the magnetic pulse and of the data stored frequency were previously defined in “Micromagnetic simulations”). In Fig. 2 we show by a color plot the imaginary part of the dynamical susceptibility as a function of both the sample frequency and the RPMA strength *K*, for the four studied values of the ring internal radii, and when the pulse is applied in the x-direction. The amplitude of the dynamic susceptibility is shown according the color scale and the dashed vertical lines separate the stable magnetic states of the ring. Note that the knot state lives between the dashed vertical lines (region II depicted in Fig. 2), while the vortex/meron states are in the left/right of the knot region, regions I and III respectively in Fig. 2a–c. Nevertheless, as discussed previously for the narrowest nanoring ($R_1 = 190\,\hbox {nm}$) there is not a meron configuration, instead for $K \ge 450\,\hbox {kJ}/\hbox {m}^3$ the ring has uniform magnetization along the *z*-direction (region IV depicted in Fig. 2d).

For the widest ring with $R_1 = 50\,\hbox {nm}$ (Fig. 2a) the vortex state (region I) shows two prominent modes, in where the main mode is non linear respect to the RPMA strength, while the second mode seems to be linear in this region. The main mode is excited close to 7.6 GHz even without RPMA ($K = 0$). While the second mode is excited close to 10 GHz and is comparable in magnitude, respect to mode 1, from RPMA values close to $250 \,\hbox {kJ}/\hbox {m}^3$. In the vortex region the resonance frequency of both modes decrease as *K* increases. Moreover, other modes can be detected but they are very small in magnitudes. This behavior change at higher *K*-values when the knot and meron states appear (region II and III). In these regions the third mode, a high frequency mode, became more intense. In region II, for the knot magnetic configuration, the resonance frequency of the two main modes increases with *K*. Finally, in region III, the resonant frequency for the main mode decrease its frequency from values of approximately above $K = 400 \,\hbox {kJ}/\hbox {m}^3$. A similar behavior holds for the ring with internal radius $R_1 = 100\,\hbox {nm}$ (Fig. 2b). Nevertheless, when the ring gets narrower with internal radius $R_1 = 160\,\hbox {nm}$ (Fig. 2c) there are only two prominent modes in the whole spectrum, and in both region II and III the resonance frequency of these modes increase with *K*. Please note that as the ring gets narrower the modes are excited at higher frequencies and the separation, in frequency, between the main modes also increases. On the other hand, for the narrowest ring with internal radius $R_1 = 190\,\hbox {nm}$ (Fig. 2d) it is possible to observe one main mode, which decrease its frequency with the anisotropy for the vortex state region. This single mode linearly increase its frequency with the RPMA in the region that correspond to the uniformly magnetized nanoring in *z*-direction (region IV).

**Figure 3:**
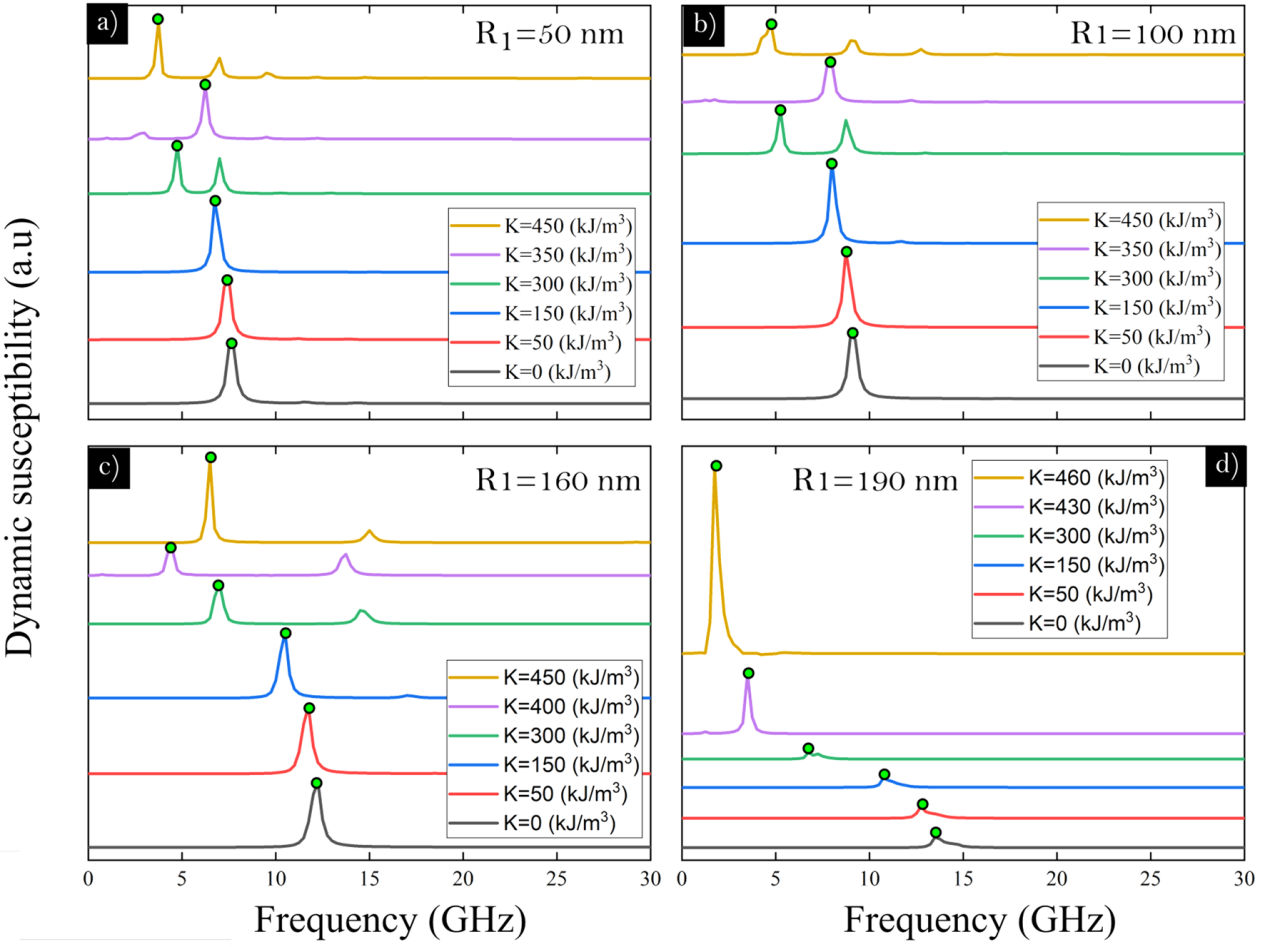
The imaginary susceptibility spectra of an isolated magnetic ring driven by the pulse in the x-direction for different values of the RPMA strength *K* and for the 4 studied values of the ring internal radius $R_1$. The main mode in each curve is denoted by a green circle).

**Figure 4:**
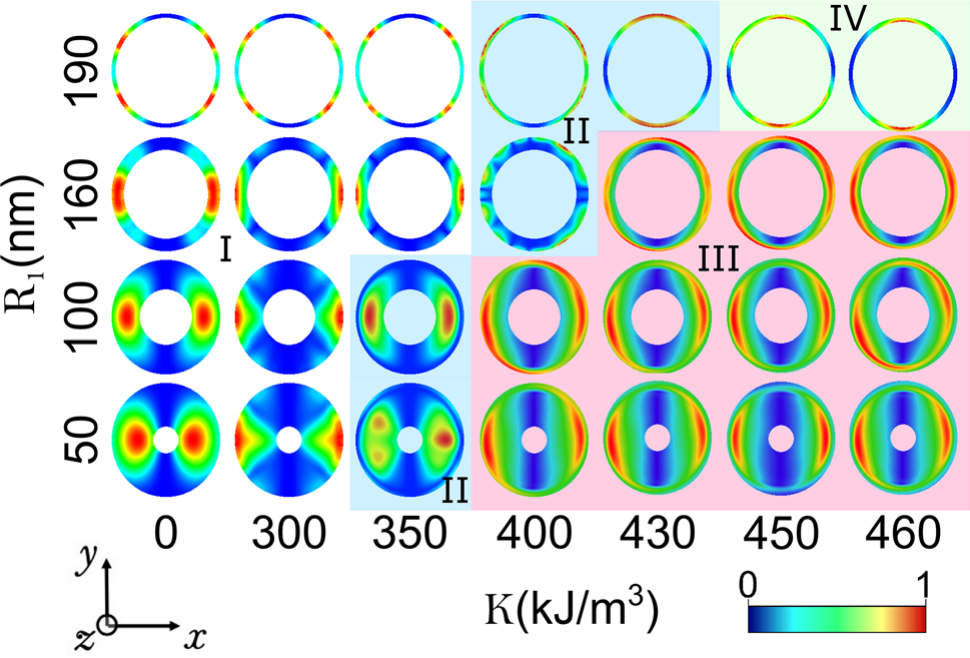
Main spatial modes of the magnetic nanoring driven by the pulse in the x-direction for different values of the RPMA strength *K* and for the 4 studied values of the ring internal radius $R_1$. Region I (white background) correspond to vortex states. Region II (blue background) correspond to knot states. Region III (red background) correspond to meron states. Region IV (green background) correspond to uniformly magnetized nanoring in *z*-direction.

In order to see in more detail the main resonance peaks of the different modes, in Fig. 3 we have plotted the imaginary part of the dynamic susceptibility as a function of the sampled frequency spectrum for the four studied ring shapes and for different RPMA strengths *K* for the same magnetic pulse. Besides, the spatial frequency distribution of oscillations of the magnetic moments for the main modes, are shown in Fig. 4 using a color scale. The spatial modes were calculated using the discrete-time Fourier transform of the magnetization. As can be seen, in the main mode, the highest oscillations of magnetic moments are distributed along the equatorial line in the pulse direction which are shifted to to *y*-direction as the ring gets narrower and the number of perturbed magnetic moments increase as *K* increases. For the vortex state the oscillations are more localized compared to the oscillations of magnetic moments in the knot and meron states, for instance note that for the meron state the pulse excites almost all the magnetic moments of the ring, except to the region close to the internal edge in the *y*-direction.

**Figure 5:**
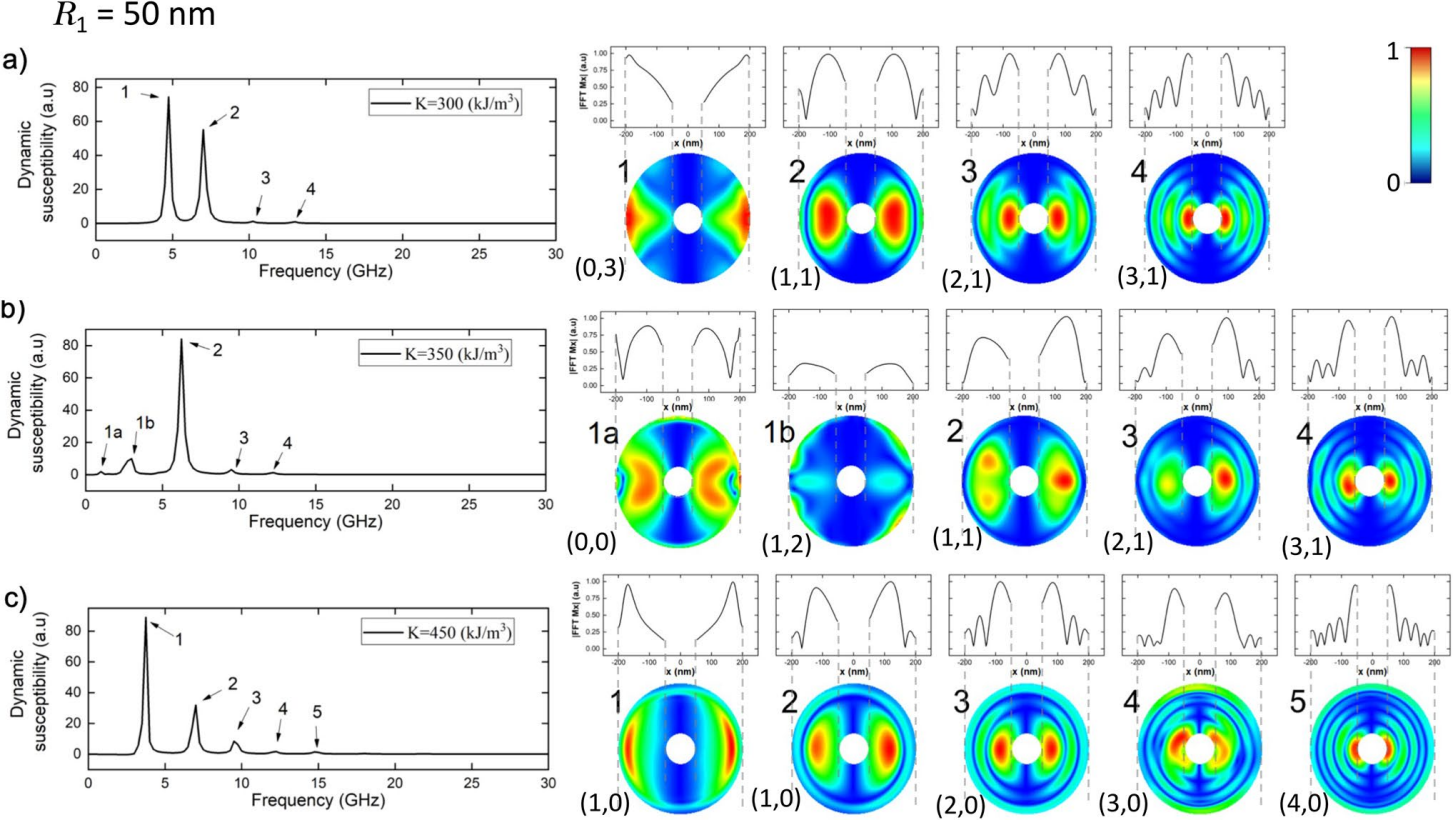
Resonance modes for the widest ring for a RPMA strength of (**a**) 300, (**b**) 350 and (**c**) $450 \,\hbox {kJ}/\hbox {m}^3$. At the left it is shown the imaginary part of the susceptibility as a function of the frequency of sample. The spatial distribution of the amplitude of resonance in the ring for each mode is depicted at the right by snapshots, while the plot above each snapshot show FFT power profile along the pulse direction in the equatorial line (*x*-direction at $y=0$). Besides, each mode is characterized in terms of number of radial and azimuthal nodes (n,m), respectively.

**Figure 6:**
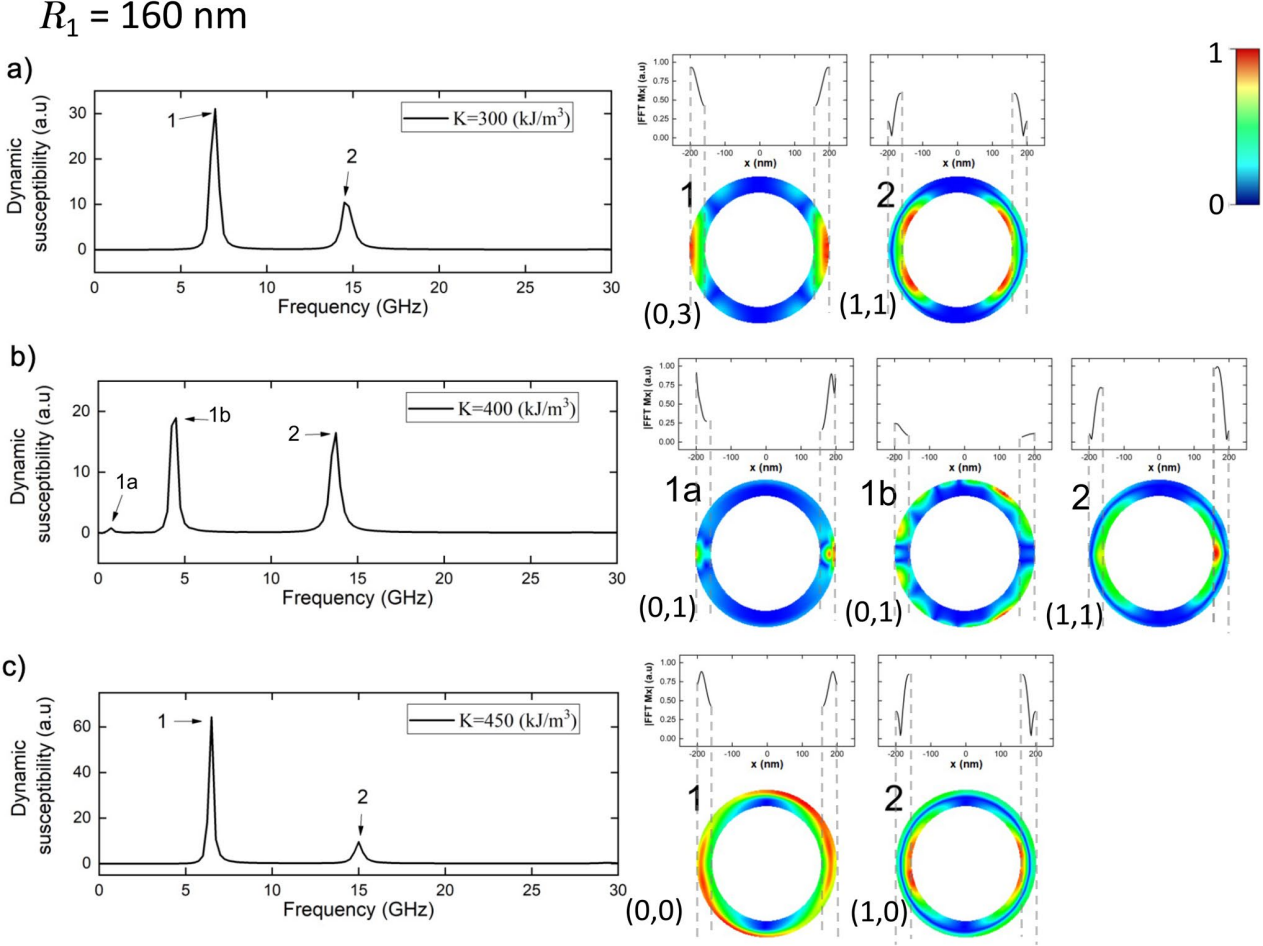
Resonance modes for the ring with internal radius $R_1 = 160\,\hbox {nm}$, for a RPMA strength of (**a**) 300, (**b**) 400 and (**c**) $450 \,\hbox {kJ}/\hbox {m}^3$. At the left it is shown the imaginary part of the susceptibility as a function of the frequency of sample. The spatial distribution of the amplitude of resonance in the ring for each mode is depicted at the right by snapshots, while the plot above each snapshot show FFT power profile along the pulse direction in the equatorial line (*x*-direction at $y=0$). Each mode is characterized in terms of number of radial and azimuthal nodes (n,m), respectively.

Moreover, as can be seen, both from Figs. 2 and 3, there are at least three modes in the spectrum. Therefore, we have also obtained the spatial modes of all other modes of Fig. 3. The spatial profiles of all modes of Fig. 3 are shown in the Supplementary Information. Here we focus on the ring of internal radii $R_1 = 50$ and 160 nm analyzing in detail all the resonant modes when the RPMA strengths are $K = 300$, 350, 450 and 300, 400, $450 \,\hbox {kJ}/\hbox {m}^3$, which are displayed in Figs. 5a–c and 6a–c respectively. We have chosen these particular values of *K* aiming to analyze in detail how the magnetic moments of the different magnetic stable configurations respond to the in plane magnetic pulse (in the *x*-direction) for each excited mode. Indeed, note that in Fig. 5a–c (or in Fig. 6a–c) the magnetic pulse is exciting a magnetic vortex, knot and meron configuration, respectively. Moreover, in order to characterize in detail all these modes, in the right on these figures, we show as snapshots the spatial profile of the magnetic moments for each mode; and above each snapshot, we show the amplitude of the FFT along the direction of the pulse in the equatorial line (at $y = 0$).

As can be seen for $R_1 = 50 \,\hbox {nm}$, when $K = 300 \,\hbox {kJ}/\hbox {m}^3$ (Fig. 5a) there exist four modes: mode 1 is the highest mode and does not show radial nodes, in this mode the maximum amplitude is located at the external edge of the vortex principally along the direction of the in plane magnetic pulse (*x*-direction) but also there are small oscillations at the external edges along two additional diametral lines. This mode is purely azimuthal with three azimuthal nodes. In mode 2 there exist two maxima of amplitude, the prominent one is located in the bulk of the vortex (distributed along the pulse direction) and the second one has lower amplitude and is excited at the external edge of the vortex (also in the equatorial line). This mode has one radial node and one azimuthal node. Mode 3 and 4 are tiny modes in amplitude, in these modes, unlike the previous ones, the magnetic moments at the external edges of the ring are not excited and have 2 and 3 maxima, respectively; distributed along the equatorial line (at y = 0). Both modes have one azimuthal node. Moreover mode 3 has two radial nodes, while mode 4 has three radial nodes. Note also, that at difference of the previous modes the main oscillation of mode 4 is at the internal edge of the ring in the equatorial line.

On the other hand, when $K = 350 \,\hbox {kJ}/\hbox {m}^3$ (Fig. 5b) the ring hosts a knot state. Now mode 2 is the highest one, and interestingly, mode 1 decreases notoriously and it is splitted in two new modes, which we call modes 1a and 1b. Note that this analysis is in agreement with Fig. 2a. Please note also that $K = 350 \,\hbox {kJ}/\hbox {m}^3$ corresponds to a knot state close to the line that separates the vortex with the knot state in Fig. 2a. Here the intensities decrease, as observed in Fig.2a, and for this reason, the intensities of the main modes change strongly, respect to $K = 300 \,\hbox {kJ}/\hbox {m}^3$. In mode 1a, the main amplitude is located in the bulk along the equatorial line. Also this mode reports oscillations at the external edge. This mode neither has radial nor azimuthal nodes. Mode 1b also shows a maximum in the bulk along the equatorial line but of lower amplitude compared to mode 1a. Moreover, in mode 1b there are small oscillations also in the external edges in two approximately perpendicular diameter lines. This mode has 2 azimuthal nodes (at difference of mode 1a in which the small oscillations are distributed along the whole external edge of the ring) and one radial node. The highest mode 2 shows a maximum in the bulk along the pulse direction, slightly deviated in the y-direction, and also reports small oscillations at the external edges. This mode has one radial node and one azimuthal node. The modes 3 and 4 at this RPMA strength show two and three maxima along the equatorial line, with main amplitudes in the bulk and in the internal edge, respectively (both slightly shifted in the y-direction). Modes 3 and 4 have both one azhimutal node and two and three radial nodes, respectively.

When $K = 450 \,\hbox {kJ}/\hbox {m}^3$ (Fig. 5c) the state corresponds to a meron configuration, at this intensity of *K*, mode 1 is the highest mode and a new mode appears, mode 5. The five modes are purely radial modes with zero azimuthal nodes. Mode 1 has main oscillations close to the external edge along the equatorial line. This mode has one radial node. Mode 2 has two maxima along the equatorial line. This mode has also one radial node. Similar to mode 2, in mode 3 the main oscillation amplitude is located at the bulk along the equatorial line. Nevertheless, mode 3 shows a new maxima respect to mode 2, showing two radial nodes. In the tiny modes 4 and 5 the main oscillations amplitude are located close to the internal edge of the ring, the modes 4 and 5 show three and four radial nodes, respectively.

It is also interesting to note that due to the RPMA, in Fig. 5a–c the main amplitude of the magnetic moments oscillations of the main modes are located at the external edge or in the bulk along the equatorial line (direction of the magnetic pulse), while in the tiny modes the main oscillation amplitude is located close to the internal edge of the ring along the pulse direction.

We continue the detailed analysis of the spatial profiles now focusing on the resonance modes due to the magnetic pulse along the *x*-direction in a narrower ring of internal radius $R_1 = 160 \,\hbox {nm}$. The corresponding modes for $K = 300\,\hbox {kJ}/\hbox {m}^3$ are shown in Fig. 6a, note that the magnetic pulse is exciting a vortex state in this case. Now only two modes can be reported; at difference for the widest ring in which 4 modes are excited as this strength of *K*. Mode 1 is the highest one, this mode is very similar as mode 1 for the widest ring (Fig. 5a), this mode has also 3 azimuthal nodes and zero radial nodes. The second mode differs respect to the case of the widest ring, note that now the main oscillations amplitude are at the internal edge of the ring along the equatorial line but shifted in the *y*-direction, also small oscillations are reported at the external edge. This mode has one radial node and one azimuthal node.

When $K = 400 \,\hbox {kJ}/\hbox {m}^3$ (Fig. 6b) there exist a new mode of resonance for the knot texture, which we call mode 1a (in agreement with Fig. 2c). In this mode the main amplitude of oscillation is close to the external edge along the equatorial lines, this mode has one azimuthal node and zero radial nodes. While for the highest mode 1b the oscillation amplitude is distributed along the external edge but distributed in the *y*-direction (note that the amplitude of the FFT spectrum along the equatorial line is small). This mode has also one azimuthal node and zero radial nodes. The mode 2 is comparable in intensity with respect to mode 1b, in this mode the main oscillation amplitude is located in the equatorial line at the internal edge of the ring. Nevertheless, also small oscillations are reported in the external edge. This mode has one radial node and one azimuthal node.

At $K = 450\,\hbox {kJ}/\hbox {m}^3$ (Fig. 6c) there are two modes of resonance in the meron state, mode 1 is the highest mode, in this mode the main oscillation amplitudes are located at the external edge in the *x*-direction but deviated also in the *y*-direction. This mode neither has radial nodes nor azimuthal nodes. In mode 2, the main oscillation amplitude is at the internal edge along the equatorial line, slightly deviated in the y-direction. Moreover there can be seen oscillation amplitude along the external edge. This mode is purely radial with one radial node and zero azimuthal nodes.

It is interesting to note that the number of radial nodes increases with the number of modes of the resonance spectra and as the ring gets narrower fewer modes are reported. Moreover the main mode in the vortex state is always a purely azimuthal mode and all the modes in the meron state have zero azimuthal nodes for an in plane magnetic pulse (see Supplementary Information 1).

Up to now we have studied in detail the effect of the RPMA on both the minimal energy magnetic configurations and on the resonance spectrum due to a magnetic pulse in the *x*-direction in nanorings with different widening. Nevertheless, as mentioned above, it is not clear if the effect of the RPMA profile can be neglected on the static and dynamic properties respect to a uniform PMA profile as the the ring gets narrower. Aiming to elucidate these issues we have also obtained the static and dynamic properties for the narrower rings of radii $R_1= 160$ and 190 nm but using a uniform PMA with uniaxial anisotropy constant $K_{{{\text {av}}}} = K/2$, for *K* up to $460 \,\hbox {kJ}/\hbox {m}^3$. Note that we choose this value for the uniform PMA constant as it corresponds to the average of the radial profile *K*(*r*) of the RPMA. The first difference comes from the minimal energy magnetic configurations (in the Supplementary Information 1, we show these magnetic configurations as snapshots). For rings with uniform PMA only the magnetic vortex and uniform magnetic configuration along *z*-direction are reported, in particular for $R_1 = 160 \,\hbox {nm}$ the ring only hosts magnetic vortex states for *K* up to $460 \,\hbox {kJ}/\hbox {m}^3$; while in the narrowest ring with uniform PMA the uniform state along *z*-direction appears for $K\ge 430\,\hbox {kJ}/\hbox {m}^3$. The absence of knot and meron states for uniform PMA rings can be explained through the torque of the effective field exerted over the magnetic moments of the ring. In the ring with uniform PMA the effective field due to the anisotropy is proportional to $K_{{{\text {av}}}} ({\mathrm{m}}\cdot {\hat{\mathrm{n}}}) {\hat{\mathrm{n}}}$, where ${\hat{\mathrm{n}}}$ is the direction perpendicular to the ring plane, this field exerts a torque over the magnetic moments which tends to align the magnetic moments perpendicularly to the ring plane, the magnitude of this torque is proportional to $K_{\text {av}}\sin {\Theta } \left| \cos {\Phi }\right|$ where $\Theta$ and $\Phi$ are the angles of the magnetization direction $\mathbf {m}$ with $d\mathbf {m}/dt$ and ${\hat{\mathrm{n}}}$, respectively. Thus the effect of changes of the $K_{\text {av}}$ value in this torque is the same in the whole ring. Therefore, as $K_{\text {av}}$ increases all magnetic moments of the ring experiment the same effect, aligning uniformly the magnetic moments towards the direction perpendicular of the ring plane. Instead, for the RPMA ring the effective field due to the anisotropy is proportional to $K(r) (\mathbf{\mathrm{m}}\cdot{\hat{\mathrm{n}}})\hat{\mathrm{n}}$. Therefore the magnitude of the torque exerted over the magnetic moments increases with the radial distance, being maximum over the magnetic moments of the external edge and zero at the internal edge. This difference of magnitude of the torque (due the effective field of anisotropy) exerted over the magnetic moments along the ring allows to stabilize the knot and meron states in the ring with RPMA.

**Figure 7:**
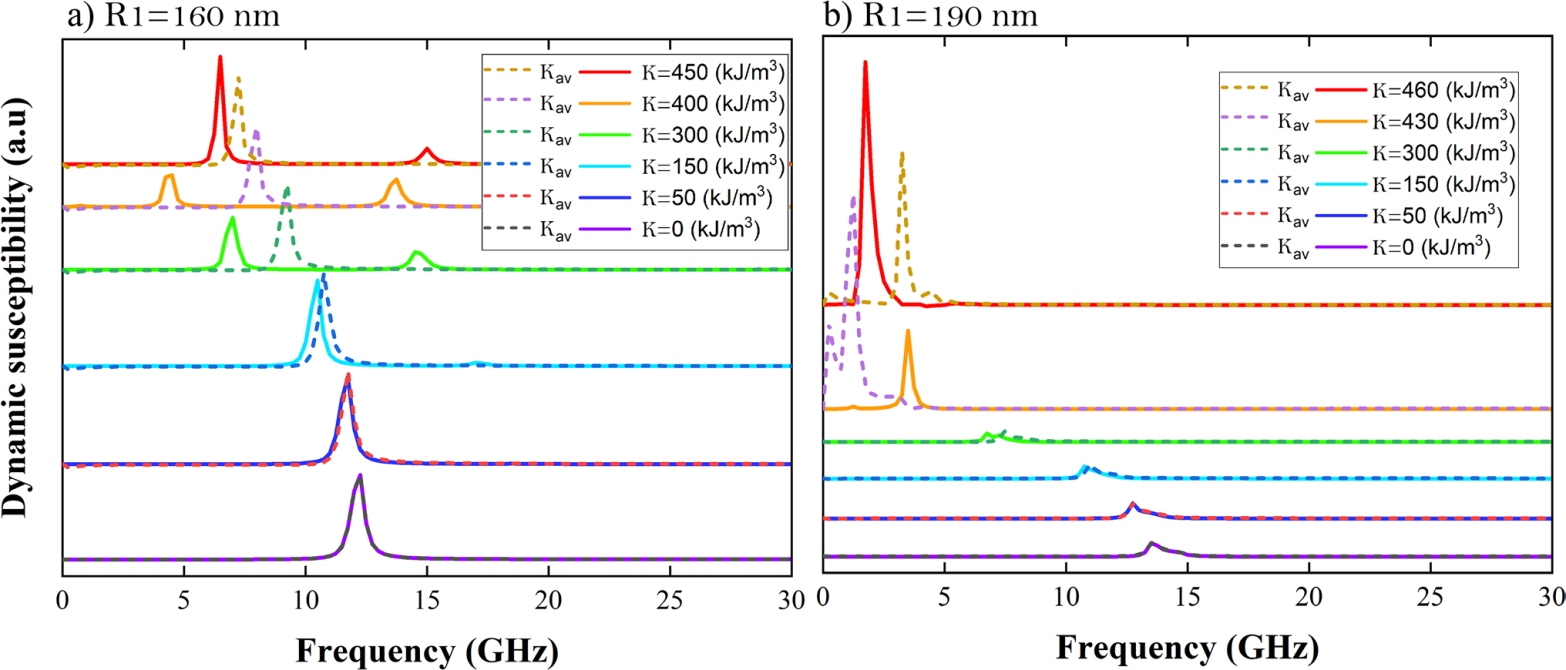
Comparison of the effect of the RPMA respect to a uniform PMA in the resonance spectra for the narrower nanorings with internal radii (**a**) $R_1 = 160$ and (**b**) $R_2 = 190$ nm due to a magnetic pulse in the *x*-direction. In these figures, the RPMA curves are shown by solid lines, while the corresponding for the uniform PMA with uniaxial anisotropy constant $K_{\text {av}}= K/2$ are depicted by dashed lines.

We continue the comparison by showing the resonance spectra in the narrower rings with $R_1 = 160$ and 190 nm in Fig. 7a,b respectively. In these figures the RPMA curves are shown by continuous lines, while the uniform PMA with the corresponding $K_{\text {av}}$ are shown in dashed lines overlapping both curves. As can be seen for the narrow ring of internal radius $R_1 = 160 \,\hbox {nm}$, Fig. 7a, the radial profile can be neglected roughly at the order of $50 \,\hbox {kJ}/\hbox {m}^3$, or below. Instead for higher values of *K* the resonance spectra change, for instance note that both the resonance frequency and the intensity of the dynamic susceptibility change between RPMA and uniform PMA. Evenmore, the second resonant mode that appear for RPMA with $K \ge 300\,\hbox {kJ}/\hbox {m}^3$, is suppressed when a uniform PMA is considered. Besides, recall that for the uniform PMA the magnetic state is always vortex while for the RPMA with $K = 400$ and $450 \,\hbox {kJ}/\hbox {m}^3$ the magnetic states correspond to a knot and a meron configuration. The difference between the spectra of resonance due to a RPMA and a uniform PMA profiles remains for the narrowest ring ($R_1 = 190\,\hbox {nm}$), Fig. 7b. For instance at $K = 150 \,\hbox {kJ}/\hbox {m}^3$ there is a smooth difference for the frequency of resonance and this difference increases with *K*. Moreover at $K = 430 \,\hbox {kJ}/\hbox {m}^3$ there is also a large difference between the intensities of the dynamic susceptibility (recall that at this value of *K* the magnetic states correspond to a knot and a uniform state in *z*-direction for the RPMA and uniform PMA, respectively). Instead, when $K \ge 450\,\hbox {kJ}/\hbox {m}^3$, the magnetic configuration is uniform along *z*-direction both for the RPMA and for the uniform PMA. Nevertheless, even if both cases shows a uniform magnetic configurations along *z*-direction, Fig. 7b shows different frequencies for the main resonant mode and different intensities of the dynamic susceptibility for both cases. This could be attributed to the fact that the pinned spins at the external edges of the ring in the RPMA have higher anisotropy than the spins at the internal edge. This is not the case for uniform PMA in where all spins, from the outer and the inner edge, are pinned with the same anisotropy strength $K_{\text {av}}$. Therefore even for the narrowest ring both the resonance spectrum and the magnetic configurations depend on the RPMA profile and on the strength of K.

**Figure 8:**
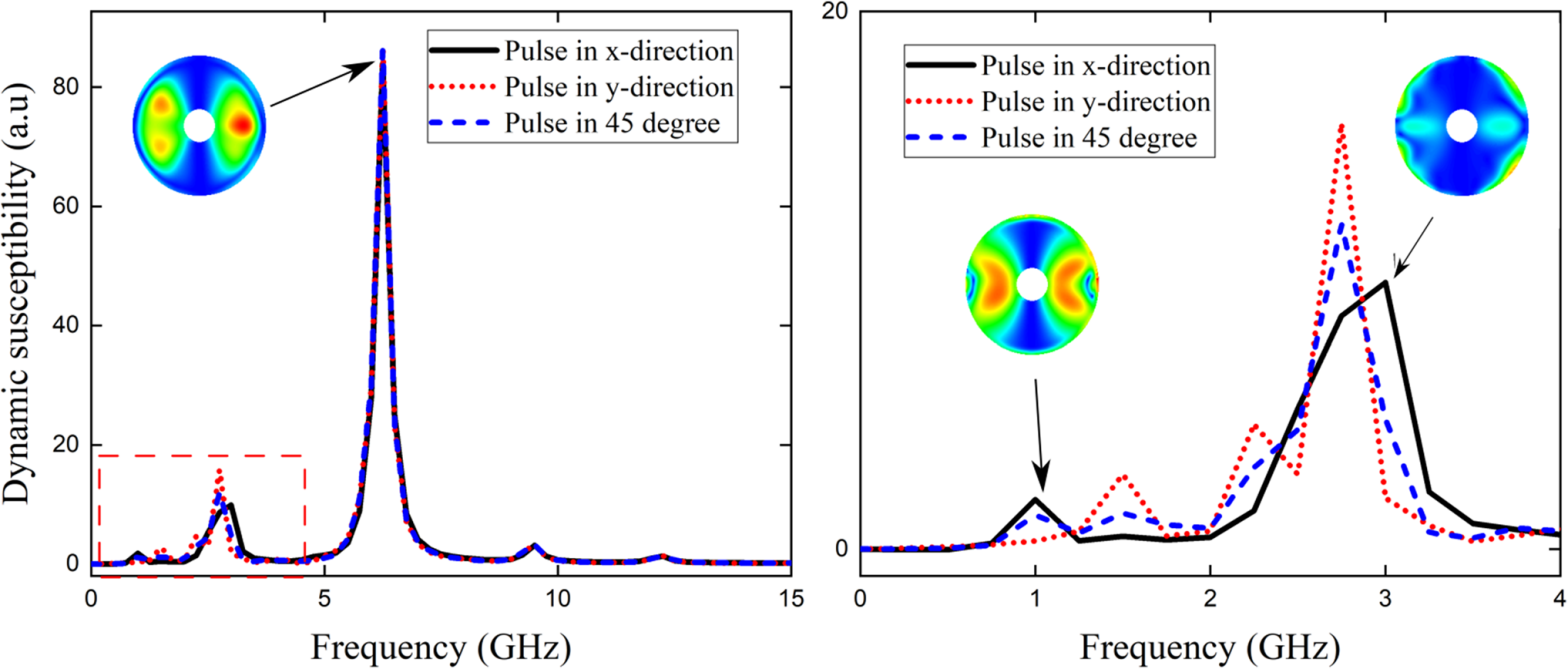
(**a**) The imaginary susceptibility spectra of the widest magnetic ring ($R_1 = 50\,\hbox {nm}$) hosting a knot state at $K= 350\,\hbox {kJ}/\hbox {m}^3$ due to the excitation of the in plane magnetic pulse at different directions. The zoom of the marked rectangle is displayed in (**b**) and the snapshots show the spatial modes of the resonant peaks.

Finally, as mentioned above as the knot state does not have azimuthal symmetry, we have also studied the effect of the direction of the in plane pulse over this magnetic state hosted in the nanoring. In Fig. 8a, we display the imaginary part of the dynamic susceptibility as a function of the sampled frequencies for the knot state hosted in the widest ring at RPMA strength $K= 350\,\hbox {kJ}/\hbox {m}^3$. The different directions of the in plane pulse are depicted by different markers in Fig. 8, full line and dotted lines correspond to the pulse along the *x*-direction and *y*-direction (respectively); while the dashed line corresponds to the pulse directed in $45^{\circ }$ in the *xy*-plane. The zoom of the dashed rectangle is shown in Fig. 8b. As can be seen the main mode does not depend on the field direction neither the other modes greater than 5 GHz. Instead, the modes below 5 GHz are sensitive to the field direction. Also, in Fig. 8b some of these modes can be turned on or turned off depending if the magnetic pulse is directed in *x* or *y*-directions and all modes are present when the field is directed in between these 2 directions, that is at $45^{\circ }$. The snapshots show the spatial mode of the resonance response of the magnetic state for the pulse in the *x*-direction.

## Conclusions

In conclusions, we have studied in detail the effect of a RPMA on static and dynamic properties of magnetic nanorings. Due to the difference of magnitude of the torque of the anisotropy effective field exerted over the magnetic moments close to the external edge respect to the magnetic moments close to the internal edge of the ring there are 3 non trivial stable magnetic states; the vortex, knot and meron states, each of these states can be controlled by the RPMA strength *K*. Moreover we have also analysed the effect of the radial anisotropy in the ferromagnetic resonance spectra for a short magnetic pulse directed through the ring plane. In the main modes, for a pulse applied in the *x*-direction, the highest oscillations of magnetic moments are distributed along the pulse direction, in the equatorial line which are shifted to the *y*-direction as the ring gets narrower. The main mode (higher peak) in the vortex configuration is always an azimuthal mode, for the four widening of the ring, in which no radial nodes can be reported. Instead, in the knot state, for the wider rings, the mode 1 decrease notoriously and split in two new modes, mode 1a and mode 1b, and mode 2 becomes the main mode. For higher values of *K* where the meron is stabilized again mode 1 is the main mode and all the modes have zero azimuthal nodes corresponding to purely radial modes or modes with neither radial nodes nor azimuthal nodes. For the wider rings with $R_1 = 50$ and 100 nm there are also secondary modes (modes 3, 4 and 5) at higher frequencies these secondary modes are very small and disappears as the ring gets narrower, also, the number of radial nodes increase with the frequency of the resonance. The modes of resonance for the different widening of the ring are sensitive to the variation of *K* and their behavior is not linear that allow us tailoring a desired set of frequencies by controlling the geometrical parameters and intrinsic parameters of the system.

On the other hand, we have also compared the effect of the RPMA over the spectrum of resonance due to the magnetic pulse on the narrower magnetic rings respect to the corresponding resonance spectrum in the magnetic rings with uniform PMA with uniaxial anisotropy constant equal to the average of the linear RPMA profile, i.e. $K_{\text {av}} = |\left\langle K(r)\right\rangle |= K/2$. Only for values of the RPMA strength *K* close and below 50 and $150 \,\hbox {kJ}/\hbox {m}^3$ both spectra are similar when $R_1= 160$ and 190 nm, respectively. Instead for higher values of the RPMA strength there are difference in the number of modes, their resonance frequencies and intensities of the dynamic susceptibility even for the narrowest ring. These differences arise not only due to the different magnetic states which are excited in the rings but also when the rings with RPMA and uniform PMA host the same magnetic texture, for instance for the vortex in the the narrow ring with $R_1 = 160\,\hbox {nm}$ at $K = 300 \,\hbox {kJ}/\hbox {m}^3$, or for the uniform magnetic states in the narrowest ring (with $R_1= 190\, \hbox {nm}$) at *K* over $450 \,\hbox {kJ}/\hbox {m}^3$. Which can be attributed to the fact that the pinned spins at the external edges of the ring in the RPMA have higher anisotropy than the spins at the internal edge. which is not the case for uniform PMA in where all spins, from the outer and the inner edge, are pinned with the same anisotropy strength $K_{\text {av}}$.

Finally we have also analysed the effect of the direction of the in plane magnetic pulse on the knot magnetic state by applying the magnetic pulse at 0, 45 and $90^{\circ }$ from the x-axis (in the *xy*-plane) showing that the resonance peaks above 5 GHz do not depend on the pulse direction while below of the main peak there are sensitive to the pulse direction. This study shows that it is possible to control both the stable magnetic configurations and the location and number of frequency peaks in FMR in nanorings with perpendicular anisotropy gradients by handling the ring aspect ratio and the RPMA strength, which could be used for potential magnetic controllable frequency devices.

## Supplementary Information

Supplementary Information.

## Data Availability

The datasets generated during and/or analysed during the current study are available from the corresponding author on reasonable request.
