# Publisher Correction: Effect of nonuniform perpendicular anisotropy in ferromagnetic resonance spectra in magnetic nanorings

**DOI:** 10.1038/s41598-021-96865-9

**Published:** 2021-08-25

**Authors:** E. Saavedra, A. Riveros, J. L. Palma

**Affiliations:** 1 Departamento de Física, Universidad de Santiago de Chile, 9170124 Santiago, Chile; 2 Escuela de Ingeniería, Universidad Central de Chile, 8330601 Santiago, Chile; 3 Center for the Development of Nanoscience and Nanotechnology (CEDENNA), 9170124 Santiago, Chile

Correction to: *Scientific Reports* 10.1038/s41598-021-93597-8, published online 09 July 2021

The original version of this Article contained an error in the Results and analysis section, under the subheading ‘Dynamic magnetic properties’, where the unitary vector “$\hat{\text{n}}$ ” did not display correctly.

This error has now been corrected in the PDF version of the Article; the HTML version was correct from the time of publication.

