# Supplementary material for: Effect of nonuniform perpendicular anisotropy in ferromagnetic resonance spectra in magnetic nanorings
Source: Sci Rep. 2021 Jul 9;11:14230. doi: 10.1038/s41598-021-93597-8 (PMC8271002; doi:10.1038/s41598-021-93597-8)
Supplement: Supplementary file 1 — Supplementary Information. [file 41598_2021_93597_MOESM1_ESM.pdf]

Supplementary Information for "Effect of nonuniform perpendicular anisotropy in ferromagnetic resonance spectra in magnetic nanorings"

E. Saavedra[1], A. Riveros[2], and J.L. Palma[2,3*]

[1]Departamento de Física, Universidad de Santiago de Chile, 9170124 Santiago, Chile
[2]Escuela de Ingeniería, Universidad Central de Chile, 8330601 Santiago, Chile
[3]Center for the Development of Nanoscience and Nanotechnology (CEDENNA), 9170124 Santiago, Chile

* juan.palma@ucentral.cl

I) SPATIAL MODE PROFILES OF MODES OF FIGURE 3

In the main text we have analyzed in detail the resonant modes for the particular case R_1 = 50 and 160 nm when the RPMA strengths are K = 300, 350, 450 and 300, 400, 450 *k*J/m^3. For completeness, here we show as snapshots all the modes of Fig.3. Classifying each of them as: (*n*, *m*), where *n*, *m* are the number of radial and azimuthal nodes, respectively

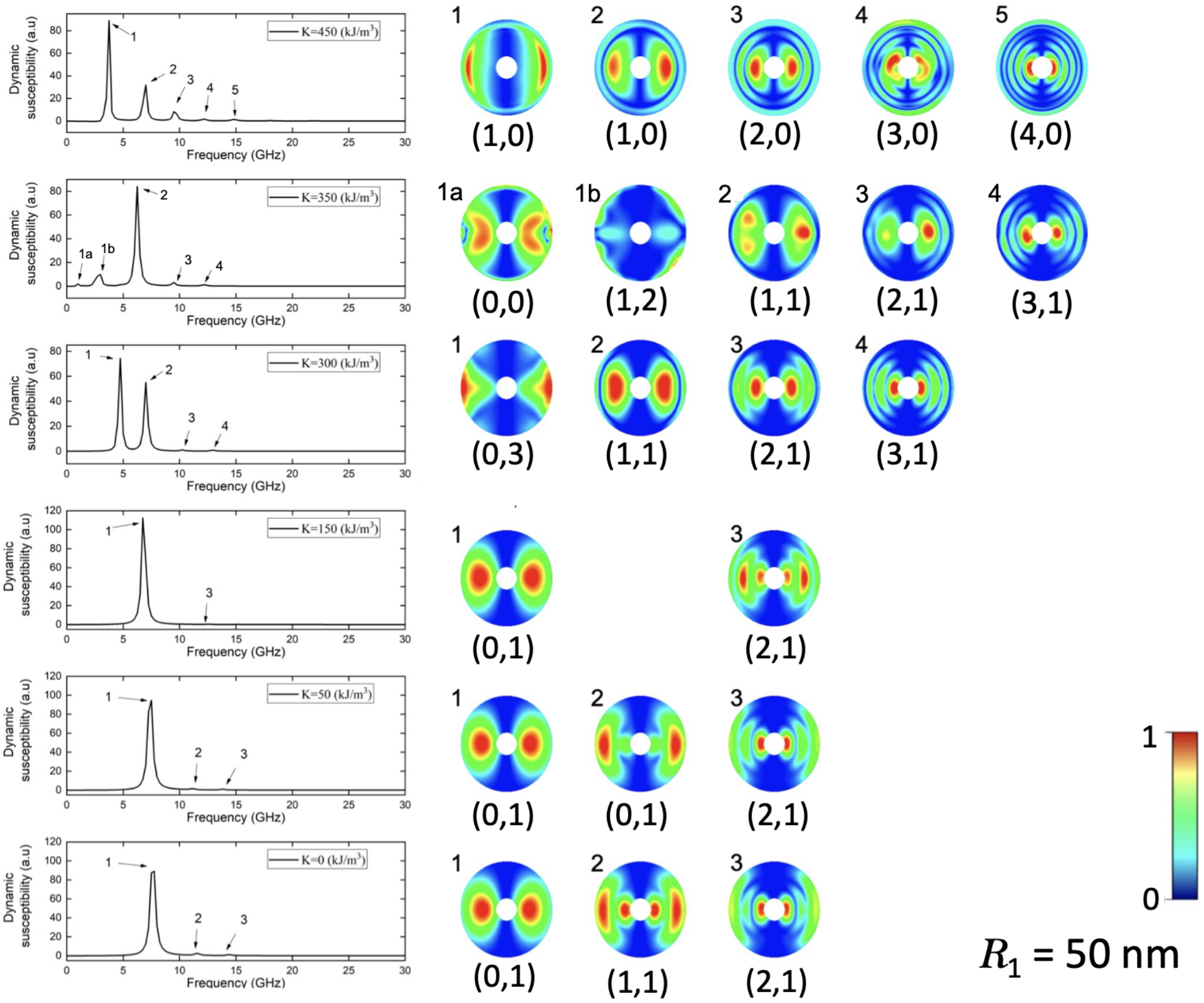

FIG. 1. (color online) Spatial mode profiles for the ring with RPMA with internal radius R_1 = 50 nm.

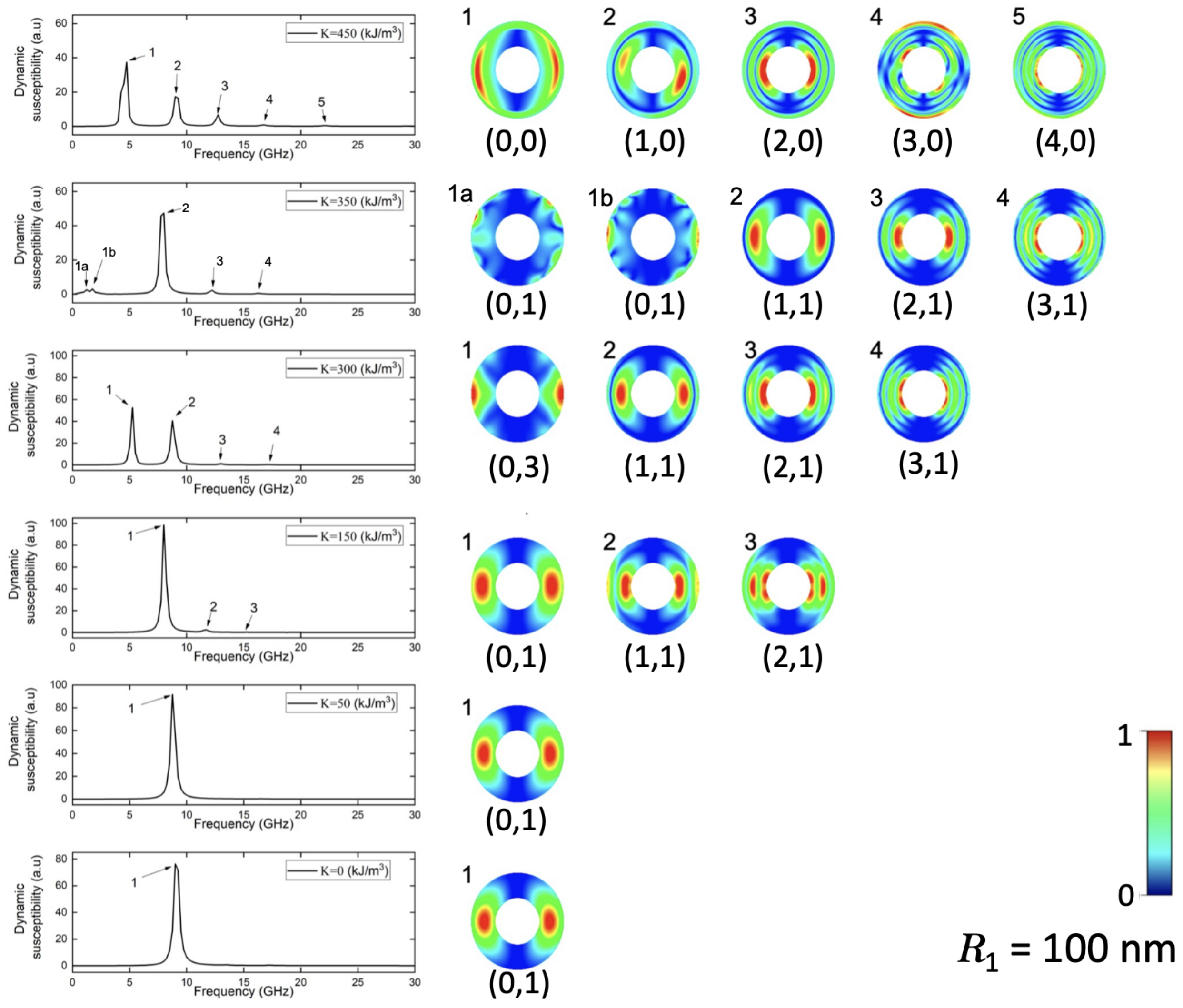

FIG. 2. (color online) Spatial mode profiles for the ring with RPMA with internal radius R_1 = 100 nm.

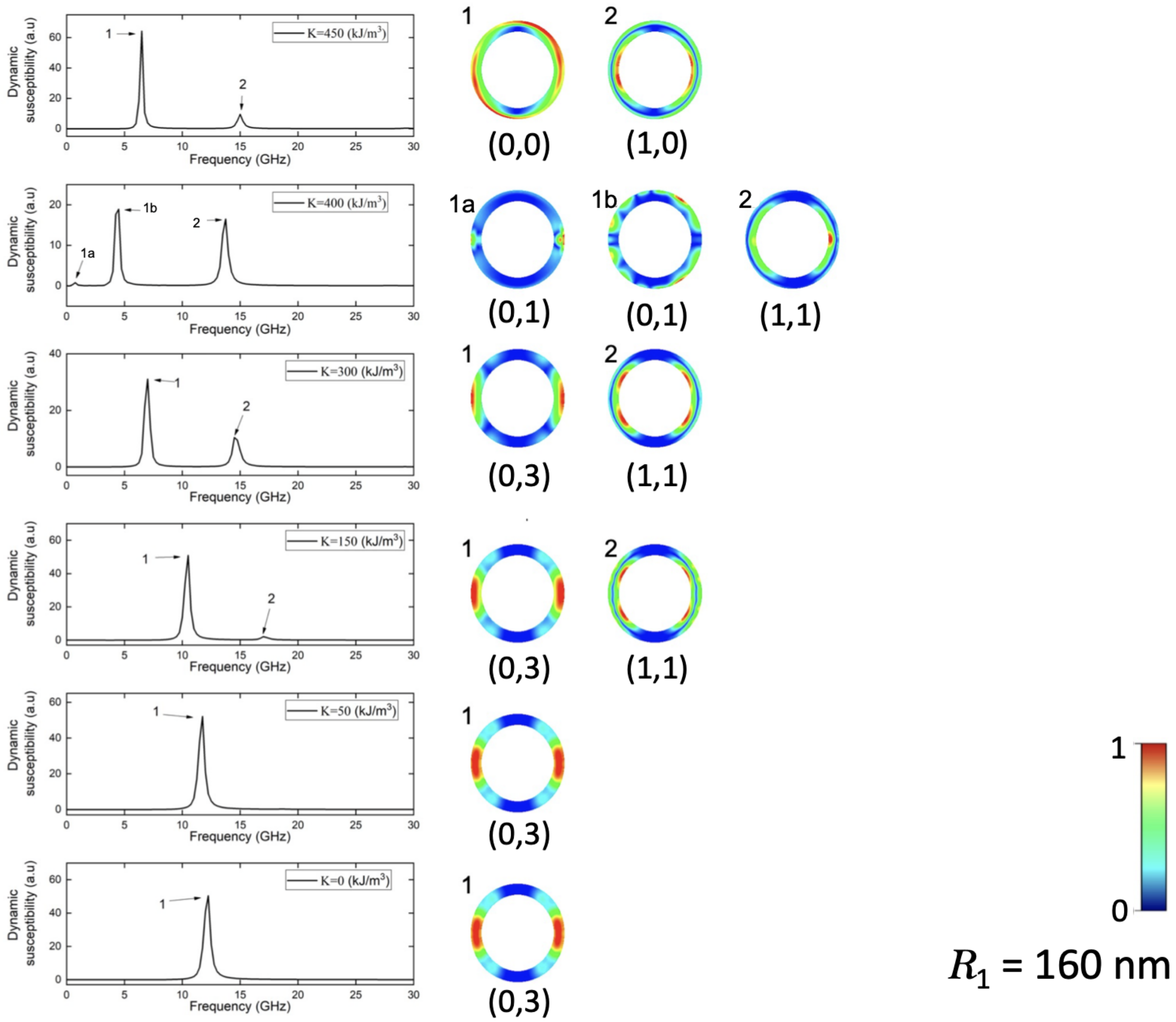

FIG. 3. (color online) Spatial mode profiles for the ring with RPMA with internal radius R_1 = 160 nm.

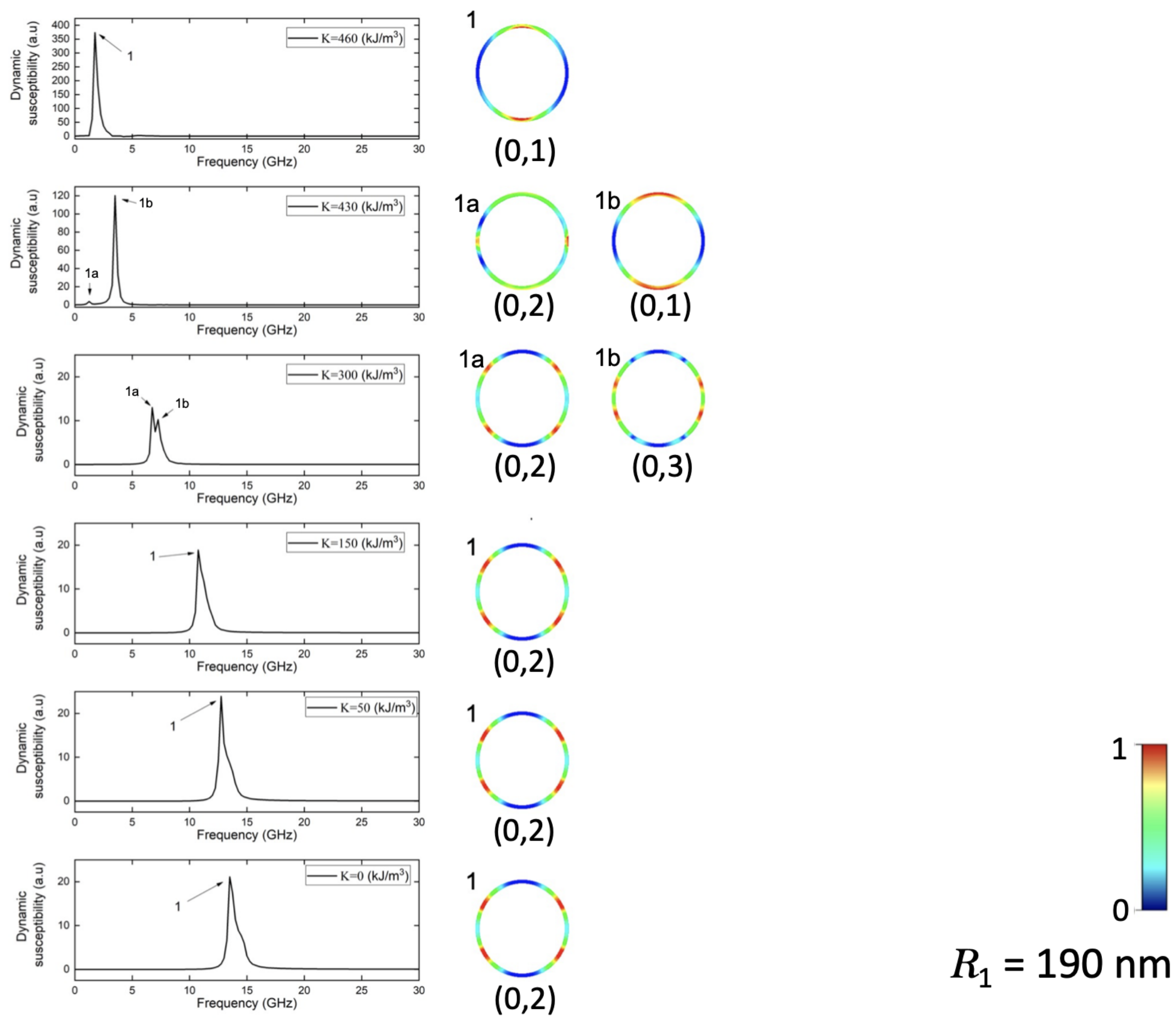

FIG. 4. (color online) Spatial mode profiles for the ring with RPMA with internal radius R_1 = 190 nm.

II) MINIMAL ENERGY MAGNETIC CONFIGURATION FOR THE NARROWER RINGS WITH UNIFORM PMA

As can be seen only the vortex and uniform magnetic state can be reported in the narrower rings with uniform PMA.

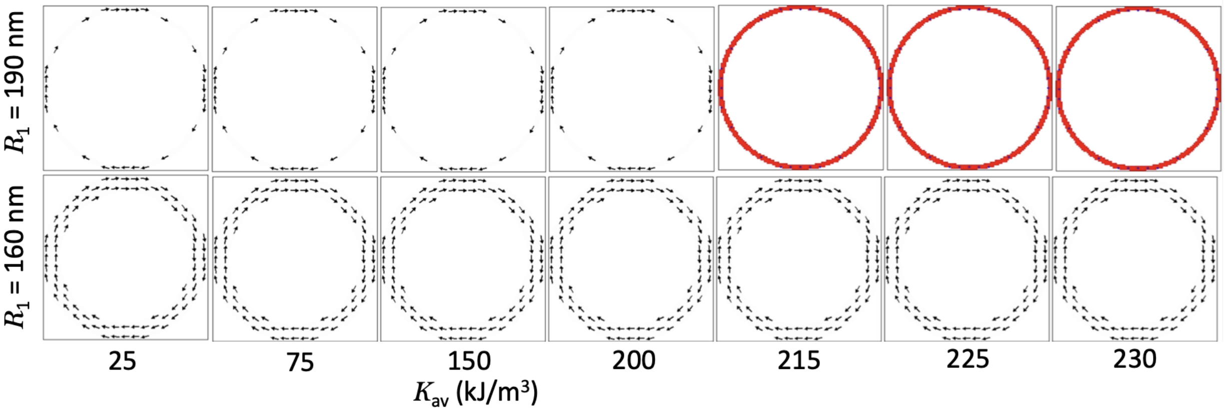

FIG. 5. *(color online) minimal energy magnetic configuration in the narrower nanorings with uniform PMA with uniaxial constant of anisotropy* K_{av}= K/2 up to K = 460 kJ/m^3.
